# Changes in the small noncoding RNA transcriptome in osteosarcoma cells

**DOI:** 10.1186/s13018-023-04362-8

**Published:** 2023-11-24

**Authors:** Hui Wang, Guiquan Cai, Fengbin Yu, De Li, Chenglong Wang, Ding Ma, Xiuguo Han, Jiajia Chen, Chuandong Wang, Jiye He

**Affiliations:** 1 Department of Orthopedic Surgery, Xinhua Hospital Affiliated to Shanghai Jiao Tong University School of Medicine (SJTUSM), Shanghai, People’s Republic of China; 2 Department of Orthopaedics, The 72nd Group Army Hospital of PLA, Huzhou, 313000 Zhejiang People’s Republic of China; 3 Department of Spine Surgery, The Second Affiliated Hospital of Nantong University, Nantong University, Nantong, 226001 Jiangsu People’s Republic of China

**Keywords:** miRNA, piRNA, snoRNA, snRNA, Repeat RNA, Osteosarcoma

## Abstract

**Background:**

Osteosarcoma has the highest incidence among bone malignant tumors and mainly occurs in adolescents and the elderly, but the pathological mechanism is still unclear, which makes early diagnosis and treatment very difficult. Bone marrow mesenchymal stem cells (BMSCs) are considered to be one of the sources of osteosarcoma cells. Therefore, a full understanding of the gene expression differences between BMSCs and osteosarcoma cells is very important to explore the pathogenesis of osteosarcoma and facilitate the early diagnosis and treatment of osteosarcoma. Small noncoding RNAs (sncRNAs) are a class of RNAs that do not encode proteins but directly play biological functions at the RNA level. SncRNAs mainly include Piwi-interacting RNAs (piRNAs), small nucleolar RNAs (snoRNAs), small nuclear RNAs (snRNAs), repeat RNAs and microRNAs (miRNAs).

**Methods:**

In this study, we compared the expression of sncRNAs in BMSCs and osteosarcoma cells by high-throughput sequencing and qPCR and looked for differentially expressed sncRNAs. CCK-8, clone formation and transwell assay were used to detect the effect of sncRNA in MG63 cells.

**Results:**

We found that 66 piRNAs were significantly upregulated and 70 piRNAs were significantly downregulated in MG63 cells. As for snoRNAs, 71 snoRNAs were significantly upregulated and 117 snoRNAs were significantly downregulated in MG63 cells. As for snRNAs, 35 snRNAs were significantly upregulated and 17 snRNAs were significantly downregulated in MG63 cells. As for repeat RNAs, 6 repeat RNAs were significantly upregulated and 7 repeat RNAs were significantly downregulated in MG63 cells. As for miRNAs, 326 miRNAs were significantly upregulated and 281 miRNAs were significantly downregulated in MG63 cells. Overexpression of piRNA DQ596225, snoRNA ENST00000364830.2, snRNA ENST00000410533.1 and miRNA hsa-miR-369-5p inhibited the proliferation and migration of MG63 cells.

**Conclusions:**

Our results provide a theoretical basis for the pathogenesis, early diagnosis and treatment of osteosarcoma.

**Supplementary Information:**

The online version contains supplementary material available at 10.1186/s13018-023-04362-8.

## Introduction

Osteosarcoma has the highest incidence among bone tumors, accounting for approximately 20% of new cases. Osteosarcoma mainly occurs in adolescents and the elderly and is highly prevalent in patients with Lefameni's disease and hereditary retinoblastoma. Osteosarcoma is a malignant tumor, but the pathological mechanism is still unclear, which makes early diagnosis and treatment very difficult. Bone marrow mesenchymal stem cells (BMSCs) were first discovered by Friedenstein in 1974 [1]. Circulating BMSCs could target small tumors and become part of the tumors [2]. BMSCs could promote the metastasis of some tumors [3, 4]. BMSCs are considered to be one of the sources of osteosarcoma cells. It has been reported that BMSCs are the progenitor cells of some sarcoma cells, such as those in bone tumors [5]. P53 activation inhibits osteoblast differentiation by inhibiting the expression of Runx2. Deletion of p53 can accelerate osteoblast differentiation but inhibits osteoblast maturation. P53 or Rb gene deletion can significantly increase the incidence of osteosarcoma in mice. Knockout of p53 or Rb in BMSCs or adipose-derived stem cells (ADSCs) resulted in the formation of smooth muscle-like tumors by MSCs in nude mice [6]. C-myc overexpression with Rb gene knockdown in human BMSCs could lead to the transformation of hBMSCs into osteosarcoma-like cells, and C-myc overexpression combined with Rb gene knockout could promote the proliferation of BMSCs and reduce the aging of BMSCs [7]. Therefore, a full understanding of the gene expression differences between BMSCs and osteosarcoma cells is very important to explore the pathogenesis and facilitate the early diagnosis and treatment of osteosarcoma.

Small noncoding RNAs (sncRNAs) are a class of RNAs that do not encode proteins but directly play biological functions at the RNA level. SncRNAs mainly include Piwi-interacting RNAs (piRNAs), small nucleolar RNAs (snoRNAs), small nuclear RNAs (snRNAs) and microRNAs (miRNAs). PiRNAs are a newly discovered type of small noncoding RNA with lengths of 24–31 nt. They play a biological roles by specifically binding to the piwi protein. Since the discovery of piRNAs, they have become a research hotspot, and related studies have made rapid progress. It has been found that piRNAs play an important role in maintaining genomic integrity, silencing transposons, tumor stem cell differentiation, epigenetic regulation and embryonic development, and disease occurrence and development. However, little is known about the specific function of piRNAs in osteosarcoma. SnoRNA was officially named in 1982, and then a series of studies were carried out [8]. Classical snoRNAs are mainly divided into three categories, including C/D box snoRNAs (SNORDs), H/ACA box snoRNAs (SNORAs) and Cajal-body specific RNAs (scaRNAs). SNORD- and SNORA-binding proteins form ribonucleoprotein (RNP) complexes, which are located in the nucleus to regulate RNA modification. Box C/D snoRNA is combined with 4 essential fibrin proteins (Nop1p, Nop56p, Nop58p and 15.5-kDa/Snu13) to form a snoRNP to guide the ribose methylation of its target. SCARNA mainly binds to the Cajal body to modify U1 and U6, while box H/ACA snoRNA can bind to the proteins Cbf5, Nop10, L7Ae and Gar1 to form snoRNPs for pseudouridine modification of target RNA. In addition, snoRNA could be stable in serum samples and easier to detect [9]. Therefore, snoRNA is expected to be a potential molecular biomarker for early diagnosis, efficacy detection and prognosis evaluation of tumors and other diseases and may provide a new target for drug therapy. SnRNAs constitute a group of small molecules in the eukaryotic nucleus and are composed of approximately 50–200 nt. They combine with related proteins to form small ribonucleoproteins (snRNPs), which mainly play an important role in processing RNA precursors and removing excess fragments (such as introns). MicroRNAs (miRNAs) include some special small ncRNAs with lengths of approximately 21–25 nt. MiRNAs play a negative regulatory role at the posttranscriptional level by promoting the degradation of target mRNAs or inhibiting the translation process. MiRNAs are closely related to the occurrence and development of tumors.

In this study, we compared the expression of sncRNAs in BMSCs and osteosarcoma cells by high-throughput sequencing and qPCR and looked for differentially expressed sncRNAs and the effect of sncRNAs on the function of MG63 cells to provide a theoretical basis for the pathogenesis, early diagnosis and treatment of osteosarcoma.

## Materials and methods

### HBMSCs isolation and culture

The project was approved by the ethics committee of Xinhua Hospital Affiliated with Shanghai Jiao Tong University School of Medicine. During joint replacement, 5 ml bone marrow blood was taken, 1 ml bone marrow blood was inoculated into a 10-cm culture dish, and 10 ml DMEM containing 10% fetal bovine serum and 1% streptomycin-penicillin solution was added to the culture dish. The cells were cultured in a cell incubator with 5% CO_2_ saturation humidity at 37 °C, and the medium was changed every 3 days.

### RNA extraction

According to the manufacturer's instructions, we added 1 ml of TRIzol reagent to each well of the 6-well culture plate, placed it on ice for lysis for 5 min, added 200 μl chloroform, let the mixture stand for 15 min, centrifuged at 12,000 rpm for 10 min, took the supernatant and placed it in a new centrifuge tube, added 500 μl isopropanol, let the mixture stand for 15 min, centrifuged at 12,000 rpm for 10 min, poured out the supernatant, retained the precipitate, added 75% ethanol solution, centrifuged at 12,000 rpm for 10 min, poured out the supernatant, retained the precipitate, air-dried the RNA precipitate, and then added DEPC water to dissolve the RNA.

### Small RNA sequencing

The original image data file obtained by high-throughput sequencing (Illumina HiSeq 2000) was transformed into the original sequencing sequence by base calling analysis. Clean data were obtained by removing the linker sequence and filtering the low-quality sequence. The preprocessed sequence data were compared with the reference genome. To comprehensively predict noncoding RNAs of different lengths, we first extracted the sequencing sequences aligned to the reference genome in each sample and then used cdhit to cluster the sequences of all samples, such as redundancy, and removed the same or similar sequences. Then, we used infernal software to search the RFAM database and detected that nonredundant sequences may contain noncoding RNA. Using the read count data obtained from the RNA expression level analysis, we quantified all the expressed RNAs in the two groups of samples to judge whether there was a significant difference in the expression between the two groups of samples. For the experiments with biological replicates, we used deseq for analysis. For experiments without biological replicates, we used edgeR for analysis. Finally, the genes with Padj less than 0.05 and a fold change greater than or equal to 2 were selected as significant differentially expressed markers.

### qPCR analysis

Total RNA was extracted with TRIzol reagent, the RNA concentration was detected by a Nanodrop 2000, and 1 μg RNA was reverse transcribed with an RNA reverse transcription kit. qPCR was carried out with the SYBR Green RT qPCR Master Mix kit according to the instructions of the kit. The reaction conditions are as follows: procedure 1: 95 °C, 30 s, 1 cycle; procedure 2: 95 °C for 5S, 60 °C for 34 s, 50 cycles; procedure 3: 95 °C 5S, 65 °C 60 s 97 °C 1 s, 1 cycle; procedure 4: 42 °C 30 s 1 cycle. The relative expression of genes was calculated according to the 2^−∆∆CT^ method, and the RNA levels were normalized to those of U6. All primers used were listed in Additional file 1: Table S1.

### Clone formation

MG63 cells were seeded into six-well cell culture plates at a density of 300 pcs/well, and after two weeks of contact culture, they were fixed with 4% PFA solution for 15 min, PBS rinsed twice, stained with 0.1% crystal violet for 10 min, and PBS rinsed three times for 5 min each.

### Transwell analysis

Cells were seeded into the upper chamber of the Transwell chamber, low serum medium was added to the lower chamber, the cells on the upper surface of the upper chamber were wiped off after 24 h of culture, the cells in the lower chamber were fixed with 4% PFA, stained with 0.1% crystal violet, and the number of cells below the upper chamber was counted after PBS washing twice.

### Statistical analysis

Excel 2017 software was used for statistical analysis, and the data are expressed as the mean ± S.D. Independent sample t tests were used to compare the mean of two groups, and one-way ANOVA was used to compare the mean of multiple groups. Differences were considered statistically significant at *P *< 0.05.

## Results

### The changes in piRNA expression in osteosarcoma cells

**Fig. 1:**
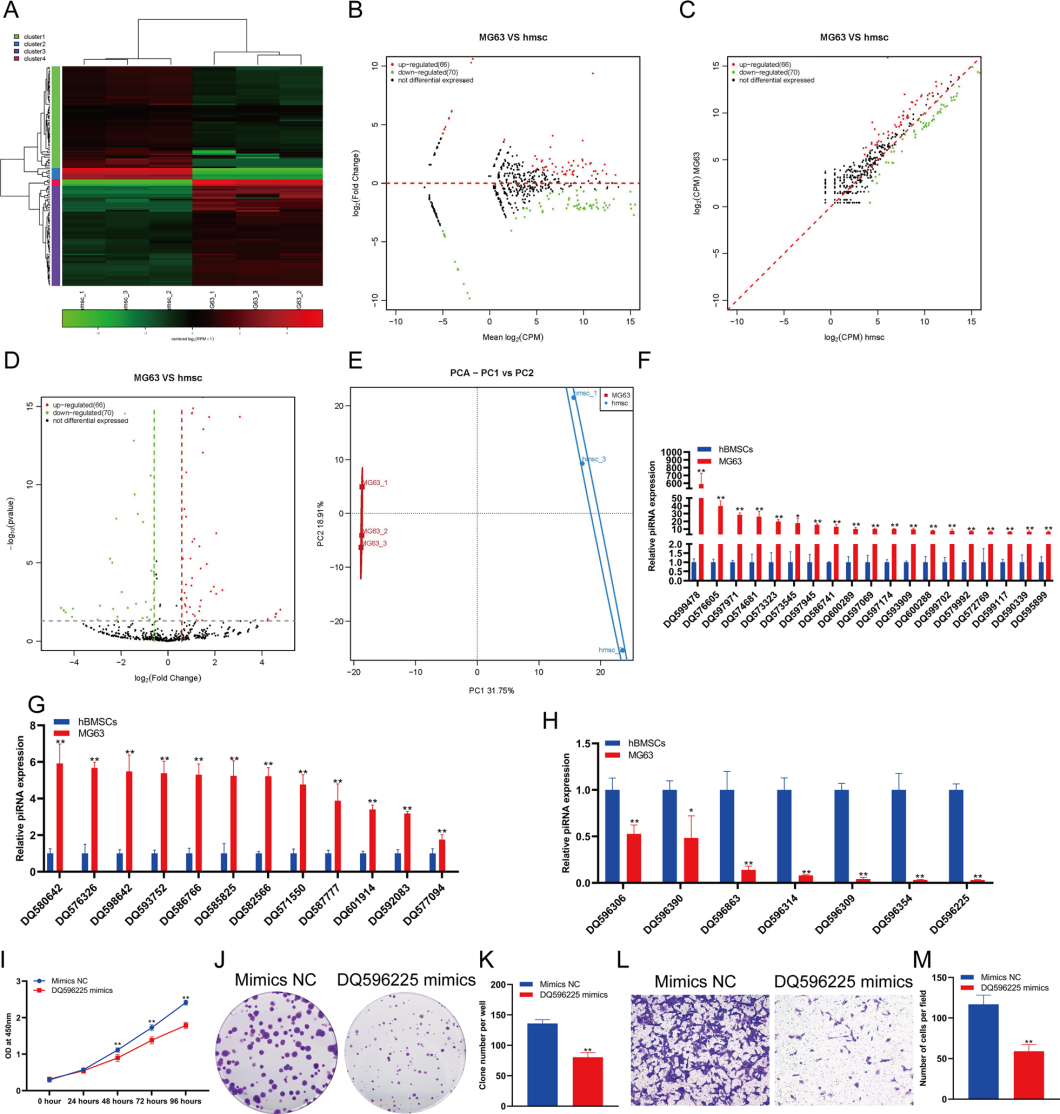
Changes in piRNA expression in osteosarcoma cells compared to hBMSCs. Heatmap (**A**), MA (**B**), scatter plot (**C**) and volcano plot (**D**) analyses of snoRNA expression in osteosarcoma cells compared to hBMSCs. **E** PCA of snoRNA expression in osteosarcoma cells compared to hBMSCs. **F** and **G** qPCR analysis showing that snoRNA expression was upregulated in osteosarcoma cells compared to hBMSCs. **H** qPCR analysis showed that snoRNA expression was downregulated in osteosarcoma cells compared to hBMSCs. **I** The proliferation of MG63 cells were detected by CCK-8. **J **The proliferation of MG63 cells were detected by clone formation. **K** Statistical analysis of the number of clones. **L** The invasion of MG63 cells were detected by transwell. **M** Statistical analysis of the number of cells. **indicates *p *< 0.01 compared to hBMSCs, and *indicates *p *< 0.05 compared to hBMSCs

Although many studies have shown the molecular function of piRNAs in the process of carcinogenesis and some assumptions and understanding have been made on the biogenesis and function of piRNAs, this research is still in a preliminary stage. First, we analyzed the expression changes in piRNA in hBMSCs and MG63 cells. The analysis results showed that there was a significant difference in the expression of piRNA between hBMSCs and MG63 cells (Fig. 1A). Through a variety of statistical analysis methods, we found that 66 piRNAs were significantly upregulated and 70 piRNAs were significantly downregulated in MG63 cells compared with hBMSCs (Fig. 1B–D). Principal component analysis (PCA) showed that MG63 cells and hBMSCs could be clearly distinguished according to the expression of piRNAs (Fig. 1E). The sequencing results were verified by qPCR. qPCR results showed that 31 piRNAs were significantly upregulated and 7 piRNAs were significantly downregulated in MG63 cells (Fig. 1F–H). The expression of piRNA DQ599478 in MG63 cells increased most significantly, with fold change of 588 in MG63 cells versus hBMSCs, while the expression of piRNA DQ596225 decreased most significantly, to approximately 3% of the level in hBMSCs (Fig. 1F–H). To detect the effect of DQ596225 on MG63 cell function, we used DQ596225 mimics to overexpress DQ596225 in MG63 cells. The results of CCK-8 (Fig. 1I)and clone formation assay (Fig. 1J, K) showed that DQ596225 overexpression could significantly inhibit the proliferation of MG63 cells. Transwell detection showed that DQ596225 overexpression could significantly inhibit the invasion of MG63 cells (Fig. 1L–M).

### The changes in snoRNA expression in osteosarcoma cells

**Fig. 2:**
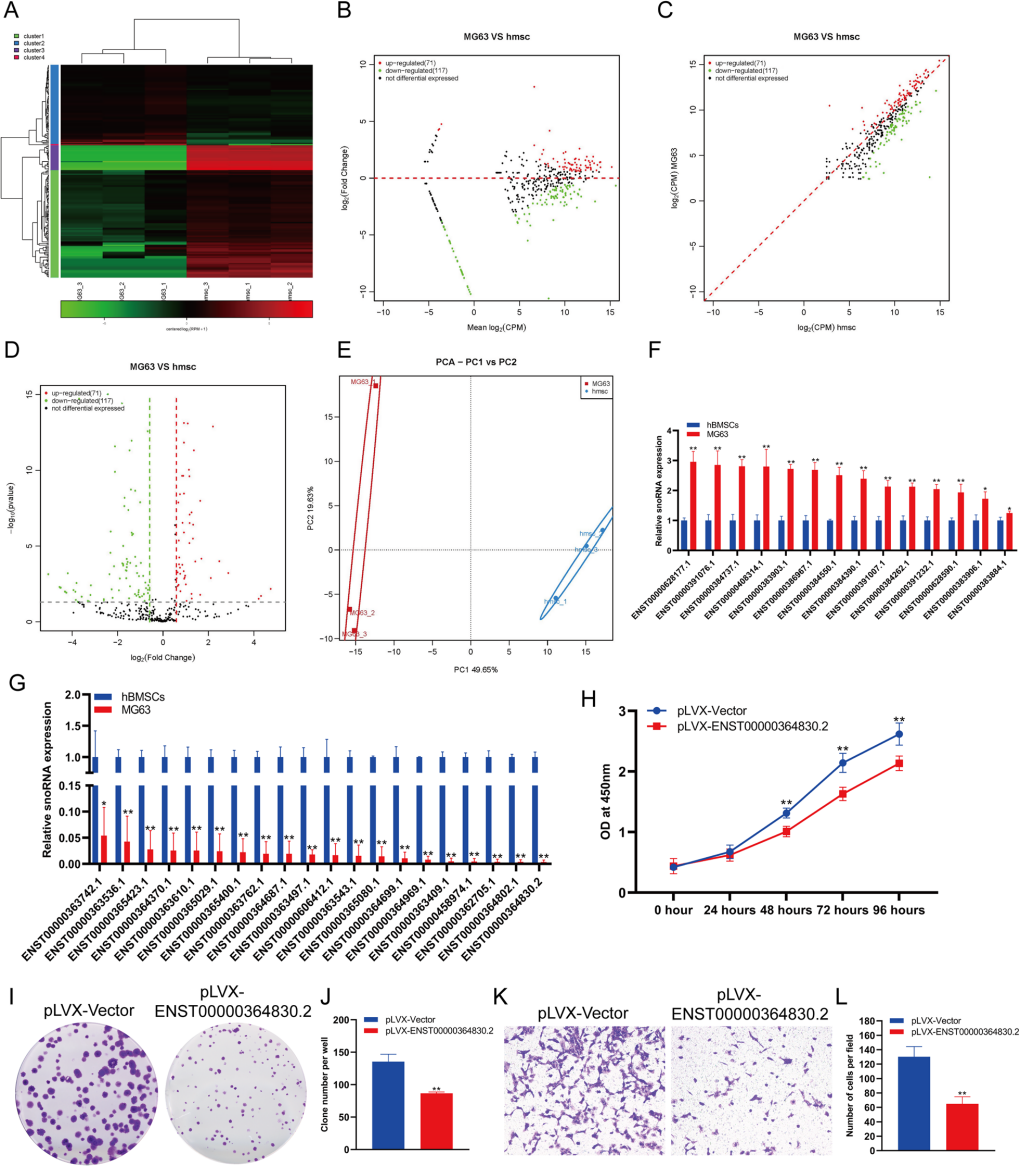
Changes in snoRNA expression in osteosarcoma cells compared to hBMSCs. Heatmap (**A**), MA (**B**), scatter plot (**C**) and volcano plot (**D**) analyses of snoRNA expression in osteosarcoma cells compared to hBMSCs. **E** PCA of snoRNA expression in osteosarcoma cells compared to hBMSCs. **F** qPCR analysis showed that snoRNA expression was upregulated in osteosarcoma cells compared to hBMSCs. **G** qPCR analysis showed that snoRNA expression was downregulated in osteosarcoma cells compared to hBMSCs. **H** The proliferation of MG63 cells were detected by CCK-8. **I** The proliferation of MG63 cells were detected by clone formation. **J **Statistical analysis of the number of clones. **K** The invasion of MG63 cells were detected by transwell. **L** Statistical analysis of the number of cells. **indicates *p *< 0.01 compared to hBMSCs, and *indicates *p *< 0.05 compared to hBMSCs

SnoRNAs have been a popular topic in recent biological research. They are encoded by introns and distributed in the nucleolus of eukaryotic cells. They have conserved structural elements and have been proven to have a variety of biological regulatory functions. We analyzed the expression changes in snoRNAs in hBMSCs and MG63 cells. The analysis results showed that there was a significant difference in the expression of snoRNAs between hBMSCs and MG63 cells (Fig. 2A). Through a variety of statistical analysis methods, we found that the expression of 71 snoRNAs was increased significantly and that the expression of 117 snoRNAs was decreased significantly in MG63 cells compared with hBMSCs (Fig. 2B–D). PCA showed that MG63 cells and hBMSCs could be clearly distinguished according to the expression of snoRNA (Fig. 1E). The sequencing results were verified by qPCR. QPCR results showed that the expression of 14 snoRNAs in MG63 cells was significantly increased, and the expression of 20 snoRNAs in MG63 cells was significantly decreased (Fig. 2F, G). The expression of snoRNA ENST00000628177.1 increased most significantly, with an increase of more than threefold in MG63 cells versus hBMSCs, while the expression of snoRNA ENST00000364830.2 decreased most significantly, to approximately 0.3% of that in hBMSCs (Fig. 2F, G). In order to detect the effect of snoRNA on MG63 cells, we overexpressed snoRNA ENST00000364830.2 in MG63 cells. The results of CCK-8 (Fig. 2H) and clone formation assay (Fig. 2I, J) showed that snoRNA ENST00000364830.2 overexpression could significantly inhibit the proliferation of MG63 cells. Transwell detection showed that snoRNA ENST00000364830.2 overexpression could significantly inhibit the invasion of MG63 cells (Fig. 1K–L).

### The changes in snRNA expression in osteosarcoma cells

**Fig. 3:**
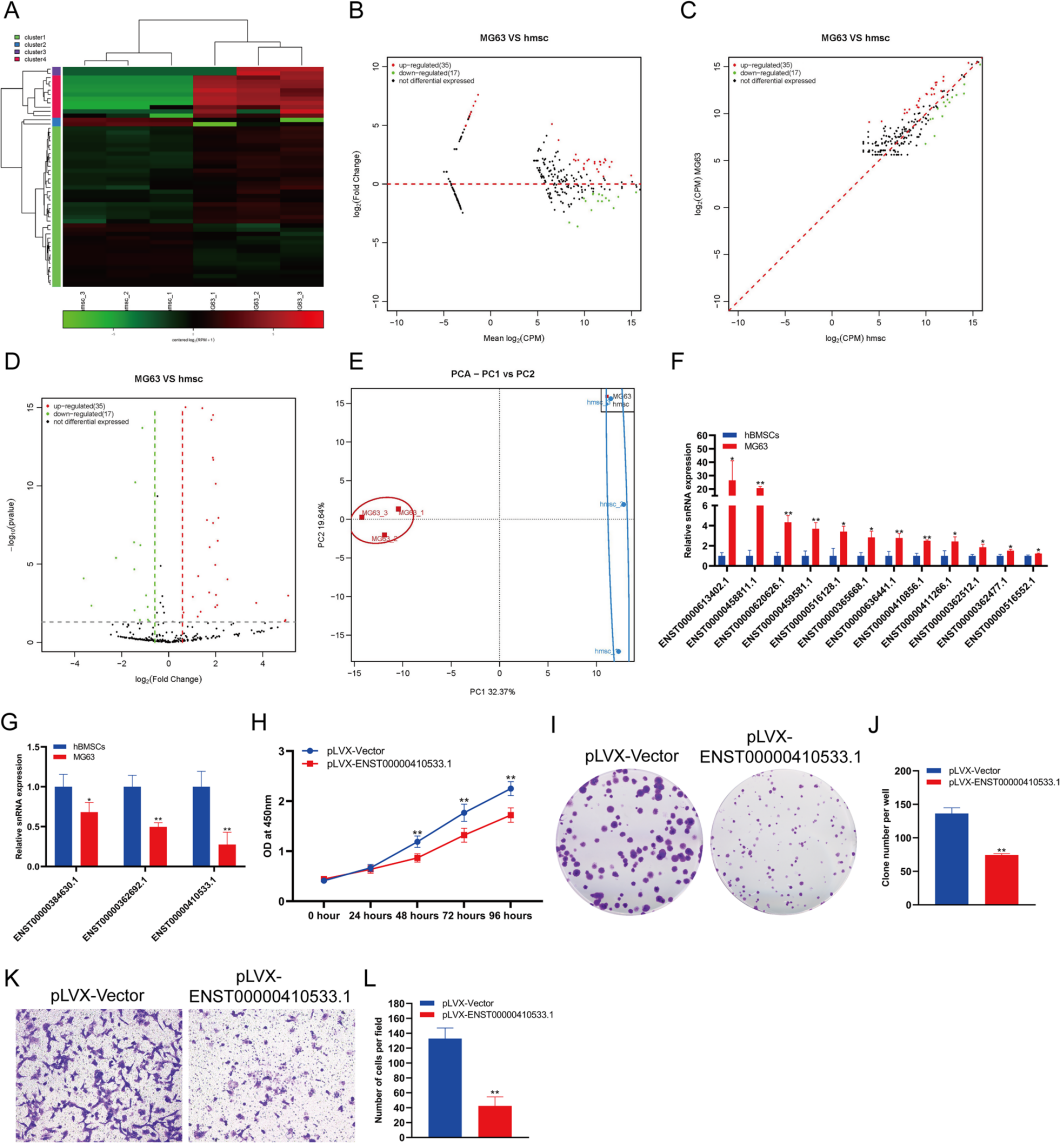
Changes in snRNA expression in osteosarcoma cells compared to hBMSCs. Heatmap (**A**), MA (**B**), scatter plot (**C**) and volcano plot (**D**) analyses of snRNA expression in osteosarcoma cells compared to hBMSCs. **E** PCA of snRNA expression in osteosarcoma cells compared to hBMSCs. **F** qPCR analysis showed that snRNA expression was upregulated in osteosarcoma cells compared to hBMSCs. **G** qPCR analysis showed that snRNA expression was downregulated in osteosarcoma cells compared to hBMSCs. **H** The proliferation of MG63 cells were detected by CCK-8. **I **The proliferation of MG63 cells were detected by clone formation. **J** Statistical analysis of the number of clones. **K** The invasion of MG63 cells were detected by transwell. **L** Statistical analysis of the number of cells. **indicates *p *< 0.01 compared to hBMSCs, and *indicates *p *< 0.05 compared to hBMSCs

SnRNAs are the main components of RNA spliceosomes in the posttranscriptional processing of eukaryotic mRNA and participate in the processing of mRNA precursors. We analyzed the expression changes in snRNA in hBMSCs and MG63 cells. The analysis results showed that there was a significant difference in the expression of snRNA between hBMSCs and MG63 cells (Fig. 3A). Through a variety of statistical analysis methods, we found that the expression of 35 snRNAs was increased significantly and the expression of 17 snRNAs was decreased significantly in MG63 cells compared with hBMSCs (Fig. 3B–D). PCA showed that MG63 cells and hBMSCs could be clearly distinguished according to the expression of snRNA (Fig. 3E). The sequencing results were verified by qPCR. The qPCR results showed that the expression of 12 snRNAs was significantly increased, and the expression of 3 snRNAs in MG63 cells was significantly decreased in MG63 cells verus hBMSCs (Fig. 3F–G). The expression of snRNA ENST00000613402.1 increased most significantly, with an increase of more than 26 times in MG63 cells verus hBMSCs, while the expression of snRNA ENST00000410533.1 decreased most significantly, to approximately 27.5% of that in hBMSCs (Fig. 3F–G). In order to detect the effect of snRNA on MG63 cells, we overexpressed snRNA ENST00000410533.1 in MG63 cells. The results of CCK-8 (Fig. 3H) and clone formation assay (Fig. 3I–J) showed that snRNA ENST00000410533.1 overexpression could significantly inhibit the proliferation of MG63 cells. Transwell detection showed that snRNA ENST00000410533.1 overexpression could significantly inhibit the invasion of MG63 cells (Fig. 3K–L).

### Changes in repeat RNA expression in osteosarcoma cells

**Fig. 4:**
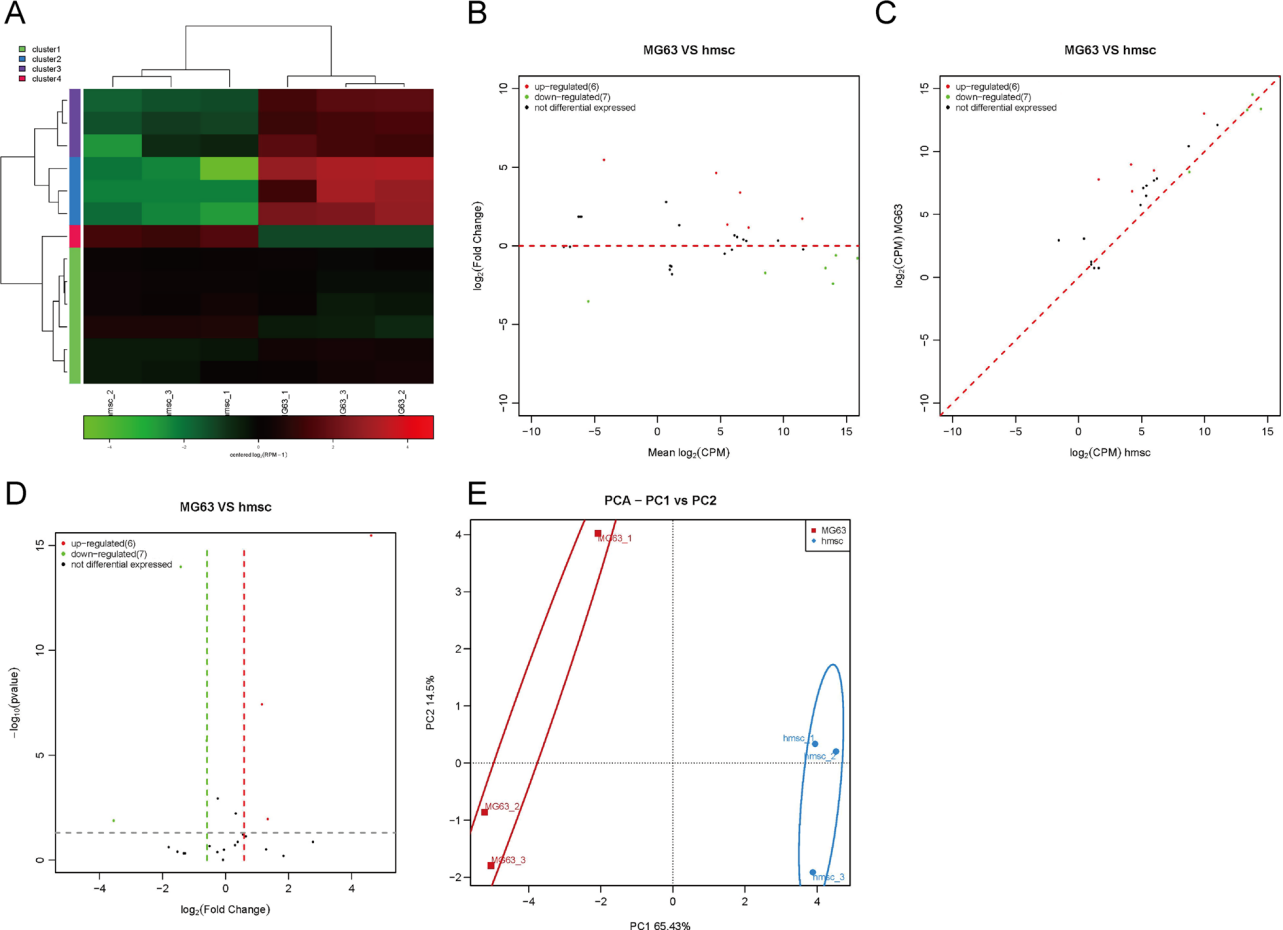
Changes in repeat RNA expression in osteosarcoma cells compared to hBMSCs. Heatmap (**A**), MA (**B**), scatter plot (**C**) and volcano plot (**D**) analyses of miRNA expression in osteosarcoma cells compared to hBMSCs. **E** PCA of miRNA expression in osteosarcoma cells compared to hBMSCs

Some RNAs with specific repeats can form secondary or tertiary structures by base complementary pairing to regulate cell function. We analyzed the expression changes in repeat RNA in hBMSCs and MG63 cells. The analysis results showed that there was a significant difference in the expression of repeat RNA between hBMSCs and MG63 cells (Fig. 4A). Through a variety of statistical analysis methods, we found that the expression of 6 repeat RNAs was significantly increased and the expression of 7 repeat RNAs was significantly decreased in MG63 cells compared with hBMSCs (Fig. 4B–D). PCA showed that MG63 cells and hBMSCs could be clearly distinguished according to the expression of repeat RNAs (Fig. 4E).

### The changes in miRNA expression in osteosarcoma cells

**Fig. 5:**
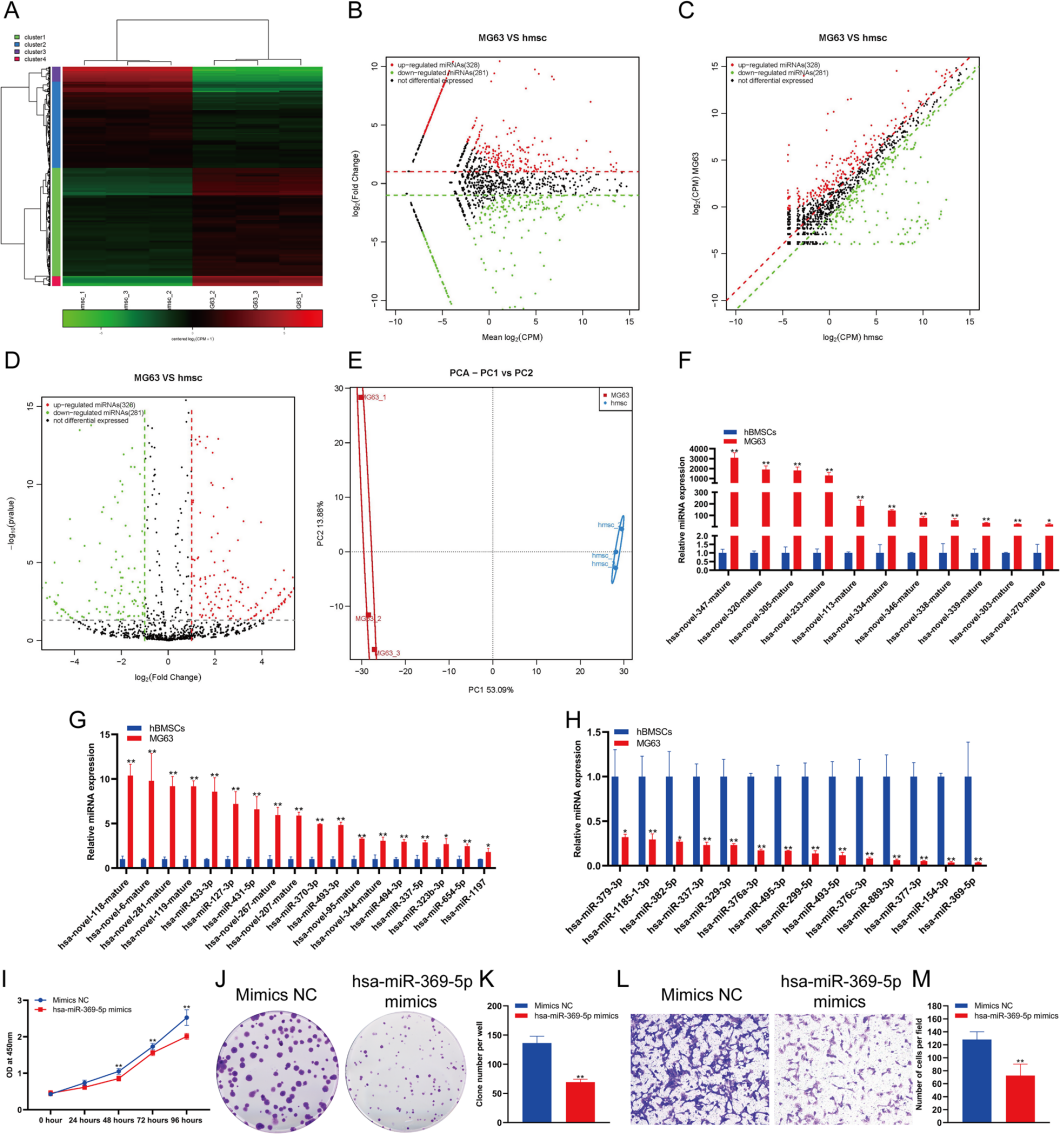
Changes in miRNA expression in osteosarcoma cells compared to hBMSCs. Heatmap (**A**), MA (**B**), scatter plot (**C**) and volcano plot (**D**) analysis of miRNA expression in osteosarcoma cells compared to hBMSCs. **E** PCA of miRNA expression in osteosarcoma cells compared to hBMSCs. **F** and **G** qPCR analysis of the upregulated expression of miRNAs in osteosarcoma cells compared to hBMSCs. **H** qPCR analysis of the expression of miRNAs downregulated in osteosarcoma cells compared to hBMSCs. **I** The proliferation of MG63 cells were detected by CCK-8. **J **The proliferation of MG63 cells were detected by clone formation. **K** Statistical analysis of the number of clones. **L** The invasion of MG63 cells were detected by transwell. **M** Statistical analysis of the number of cells. **indicates *p *< 0.01 compared to hBMSCs, and *indicates *p *< 0.05 compared to hBMSCs

MiRNAs play an important role in posttranscriptional regulation and play an important role in the occurrence and development of diseases. We analyzed the expression changes in miRNA in hBMSCs and MG63 cells. The analysis results showed that there was a significant difference in the expression of miRNA between hBMSCs and MG63 cells (Fig. 5A). Through a variety of statistical analysis methods, we found that the expression of 326 miRNAs was significantly increased and the expression of 281 miRNAs was significantly decreased in MG63 cells compared with hBMSCs (Fig. 5B–D). PCA showed that MG63 cells and hBMSCs could be clearly distinguished according to the expression of miRNA (Fig. 5E). The sequencing results were verified by qPCR. The qPCR results showed that the expression of 29 miRNAs in MG63 cells was significantly increased, and the expression of 14 miRNAs in MG63 cells was significantly decreased (Fig. 5F–H). The expression of hsa-novel-347-mature cells increased most significantly, with an increase in more than 3088 times in MG63 versus hBMSCs, while the expression of hsa-miR-369-5p decreased most significantly, to approximately 0.35% of that in hBMSCs (Fig. 5F–H). We also found that there were significant differences in the expression levels of 19 novel miRNAs between hBMSCs and MG63 cells (Fig. 5F–H). To detect the effect of hsa-miR-369-5p on MG63 cell function, we used hsa-miR-369-5p mimics to overexpress hsa-miR-369-5p in MG63 cells. The results of CCK-8 (Fig. 5I) and clone formation assay (Fig. 5J–K) showed that hsa-miR-369-5p overexpression could significantly inhibit the proliferation of MG63 cells. Transwell detection showed that hsa-miR-369-5p overexpression could significantly inhibit the invasion of MG63 cells (Fig. 5L–M).

## Discussion

The pathogenesis mechanism of osteosarcoma is not completely clear, and it is considered to be a disease caused by many factors. For example, gene deletion or mutation may lead to osteosarcoma. P53 and Rb gene mutations are the most common in osteosarcoma. Nearly 50% of osteosarcoma patients have p53 gene deletions or mutations, and approximately 70% of osteosarcoma patients have Rb gene mutations. Therefore, the p53 and Rb genes are considered to be directly related to the occurrence of osteosarcoma [10]. The bone marrow and cell microenvironment play essential supporting roles in the occurrence of osteosarcoma. Previous studies have indicated that MSCs and their differentiated progenitor cells are an important source of soft tissue sarcoma and primary bone tumor cells. In recent years, osteosarcoma has been considered a differentiation defect disease caused by genetic or epigenetic damage during osteogenic differentiation. In osteosarcoma, tumor cells are similar to osteoblasts, showing symptoms of osteoblast differentiation with malignant osteoids. There are not only osteoblast areas but also chondroblast or fibroblast areas. This feature suggests that osteosarcoma cells may originate from MSCs [11]. However, the specific stage of osteogenic differentiation of tumor cells derived from BMSCs is still difficult to determine [12]. Therefore, mastering the difference in gene expression between osteosarcoma cells and BMSCs is very important to understand the pathogenesis of osteosarcoma. The osteogenic differentiation of osteosarcoma cells is considered to be a new method for the treatment of osteosarcoma. Although differentiation therapy cannot kill tumor cells, it can cause tumor cells to lose their self-renewal and proliferation abilities by inducing terminal differentiation of osteosarcoma cells. At the same time, it can also avoid some toxicity and chemoresistance related to the current treatments for osteosarcoma. Therefore, differentiation therapy has gradually become a research hotspot in the treatment of osteosarcoma. At present, there are few studies on the gene expression difference between osteosarcoma and BMSCs, and there is still a lack of research on osteosarcoma cancer stem cell differentiation therapy.

PiRNA specifically binds to piwi subfamily proteins in the Argonaut protein family, which can guide the piwi protein and its related epigenetic mechanism to program the genome or transcriptome by identifying a large number of piRNA complementary sequences, resulting in transcriptional silencing of specific target genes while helping to maintain DNA integrity and tumor stem cell differentiation, epigenetic regulation and embryonic development and disease occurrence and development [13, 14]. Studies have reported that piRNA-piR651 is overexpressed in several types of human cancer tissues compared with paired adjacent normal tissues, including gastric cancer, lung cancer, mesothelial carcinoma, cervical cancer, colon cancer, liver cancer, breast cancer and multiple myeloma tissue [13, 15, 16]. These findings suggest that piR651 may play a carcinogenic role in carcinogenesis. PiRNA has been widely studied because of its role in tumor biology. In recent years, with the deepening of research, whether piRNAs and piwi proteins can become prognostic biomarkers of tumor-related diseases has also been actively explored, and an increasing number of biological functions, such as piRNAs, can provide opportunities to improve the clinical diagnosis and treatment of tumor diseases. In this study, we found that the expression of 31 piRNAs increased and that the expression of 7 piRNAs decreased in MG63 cells versus hBMSCs. At present, no study has reported the regulatory effect of piRNAs on osteosarcoma cells. In this study, we found the role of piRNA in regulating MG63 cell function.

SnoRNAs are traditionally considered to be involved in pseudouridine and 2'-methylation modification during ribosomal RNA (rRNA) transcription. Recently, box C/D snoRNAs that do not recognize the targeted sequence via base pairing have also been found, and these are called orphan snoRNAs. In vertebrates, most of them are encoded by intron regions, and each intron encodes no more than 1 snoRNA [17]. Early and effective diagnosis can significantly prolong the survival time of tumor patients. The time of tumor discovery determines the treatment mode and prognosis of patients. SnoRNAs are closely related to the occurrence and development of tumors. They exist stably in high abundance in blood samples. They have the basic characteristics of early diagnostic molecular markers and have the potential to be used as a large-scale physical examination index. SnoRNAs are biomarkers for the early screening of colorectal cancer. SNORA21 and SNORA42 could promote the occurrence and development of colorectal cancer and can be used as biomarkers for predicting the prognosis of colorectal cancer [18–20]. The expression of SNORA42 is negatively correlated with the survival of patients with non-small-cell lung cancer (*P *< 0.05). The expression level of SNORA42 is higher in CD133^+^ non-small-cell lung cancer cells than in the corresponding normal control cells. Knockout of SNORA42 decreased the proliferation and self-renewal ability of tumor-initiating cells in vitro, but the high expression of SNORA42 in nontumor cells could enhance the proliferation and self-renewal ability of cells [21]. It has been reported that the snoRNA host genes SNHG4, SNHG1, SNHG16, SNHG22, SNHG8 and SNHG6 are involved in the regulation of osteosarcoma function [22–26]. It has also been reported that SNORD3A, SNORA13 and SNORA28 are involved in the regulation of doxorubicin resistance in osteosarcoma cells [27]. In this study, we found that 14 snoRNAs were upregulated in MG63 cells, while 20 snoRNAs were downregulated in MG63 cells. We found that snoRNA ENST00000364830.2 significantly inhibited the proliferation and invasion of MG63 cells. The regulatory effect of snoRNA on osteosarcoma cell function and the related mechanism need to be further studied.

Pre-mRNA splicing is an important part of eukaryotic gene expression and is catalyzed by spliceosomes. SnRNAs are important structural and functional components of spliceosomes. The splicing of pre-mRNA is an important posttranscriptional processing step for eukaryotic genes. The splicing of pre-mRNA regulates a variety of life activities, such as tumorigenesis, development, and neural activity. Many studies have shown that splicing abnormalities are associated with a variety of diseases. SnRNA is neither a precursor of any other RNA nor an intermediate product of other RNA metabolism but an independent entity with unique functions. It has been reported that U1 snRNP is involved in regulating tumor migration and invasion in vitro [28]. In our study, we found that the expression of 12 snRNA fragments increased in MG63 cells, while the expression of 3 snRNA fragments decreased in MG63 cells. At present, no research has reported the regulatory effect of snRNA on the function of osteosarcoma cells. We found that snRNA ENST00000410533.1 significantly inhibited the proliferation and invasion of MG63 cells. We will further study the regulatory effect of these differentially expressed snRNAs on the function of osteosarcoma cells in future research.

RNA tends to form secondary or tertiary structures through complementary base pairing in cells. The expression changes in RNA containing special repeats in cells will regulate cell function [29, 30]. In this study, we found that there were significant changes in the expression of 13 repeat RNAs in BMSCs and MG63 cells. However, the effect of these repeat RNAs on the function of osteosarcoma cells has not been reported.

MiRNAs are endogenous small RNAs with lengths of 19–24 nt. They can target the 3' untranslated region of related mRNA through incomplete pairing, guide the formation of silencing complexes, hinder the translation of and even degrade target gene mRNA to regulate the expression of related proteins. In this study, we found that 29 miRNAs were highly expressed in MG63 cells and that 14 miRNAs were expressed at low levels in MG63 cells. We found that the expression of miR-369-5p, miR-154-3p and miR-337-3p decreased significantly in MG63 cells versus hBMSCs. It has been reported that hsa-miR-369-5p regulates the function of gastric cancer cells as a tumor suppressor gene [31] and participates in the regulation of adipogenic differentiation of BMSCs [32]. MiR-154-3p can inhibit the growth of thyroid cancer [33]. MiR-337-3p is involved in inhibiting the function of gastric cancer, epithelioid ovarian cancer, liver cancer, clear cell renal cell carcinoma and cervical cancer [34–37]. We found that hsa-miR-369-5p could significantly inhibit the proliferation and invasion of MG63 cells. The above results are consistent with our assumption, and we found the pathogenic genes of osteosarcoma by comparing the expression differences in sncRNA between BMSCs and osteosarcoma cells.

## Conclusion

HBMSCs are considered to be one of the origins of osteosarcoma, but the difference in gene expression between hBMSCs and osteosarcoma cells is unclear. Small RNA is essential for the regulation of cell function. Using high-throughput sequencing and qPCR detection, we found that there were significant differences in the expression of piRNA, snoRNA, snRNA, repeat RNA and miRNA between hBMSCs and MG63 cells. This study provides important targets for the early diagnosis and treatment of osteosarcoma.

### Supplementary Information

**Additional file 1.** Table S1.

## Abbreviations

piRNAs: Piwi-interacting RNAs
snoRNAs: Small nucleolar RNAs
snRNAs: Small nuclear RNAs
miRNAs: MicroRNAs
